# Role of integrin alpha8 in murine model of lung fibrosis

**DOI:** 10.1371/journal.pone.0197937

**Published:** 2018-05-29

**Authors:** Chi F. Hung, Carole L. Wilson, Yu-Hua Chow, Lynn M. Schnapp

**Affiliations:** 1 Division of Pulmonary, Critical Care, and Sleep Medicine, University of Washington, Seattle, Washington, United States of America; 2 Division of Pulmonary, Critical Care, Allergy and Sleep Medicine, Medical University of South Carolina, Charleston, South Carolina, United States of America; Centre National de la Recherche Scientifique, FRANCE

## Abstract

**Background:**

Integrin α8 (ITGA8) heterodimerizes with integrin β1 and is highly expressed in stromal cells of the lung. Platelet-derived growth factor receptor beta (PDGFRβ+) cells constitute a major population of contractile myofibroblasts in the lung following bleomycin-induced fibrosis. Integrin α8β1 is upregulated in fibrotic foci in bleomycin-induced lung injury. However, the functional role of ITGA8 in fibrogenesis has not been characterized. In this study, we examined whether genetic deletion of ITGA8 from PDGFRβ+ cells in the lung altered fibrosis.

**Methods:**

Pdgfrb-Cre/+;*Itga8*^*flox/-*^ or Pdgfrb-Cre/+;*Itga8*^*flox/flox*^ (Cre+) and control mice (Cre-) were used for *in vitro* and *in vivo* studies. Primary cultures of PDGFRβ+ cells were exposed to TGFβ, followed by RNA isolation for qPCR. For *in vivo* studies, Cre+ and Cre- mice were characterized at baseline and after bleomycin-induced fibrosis.

**Results:**

PDGFRβ-selected cells from Cre+ animals showed higher levels of *Col1a1* expression after treatment with TGFβ. However, Cre- and Cre+ animals showed no significant difference in measures of acute lung injury or fibrosis following bleomycin challenge.

**Conclusion:**

While ITGA8 deletion in lung PDGFRβ+ stromal cells showed evidence of greater *Col1a1* mRNA expression after TGFβ treatment *in vitro*, no functional difference was detected *in vivo*.

## Introduction

Integrin α8 (ITGA8) is a cell surface protein that belongs to the alpha integrin family of transmembrane cell surface receptors. Similar to other alpha integrins, ITGA8 requires formation of a heterodimer with β1 integrin for receptor function [1]. Contractile stromal cells in multiple organs express ITGA8 and its expression is critical in nephrogenesis [1, 2]. In the lung, ITGA8 expression is especially enriched in interstitial stromal cells following bleomycin-induced fibrosis [3].

There is significant interest in integrins as targets for anti-fibrotic therapy recently due to their role in fibrosis. Integrins may regulate cell migration, proliferation, apoptosis, and in some cases, may be important in activating latent TGFβ, a key profibrotic cytokine. Studies have shown that integrin αv-containing integrins (such as αvβ1, αvβ3 αvβ5, αvβ6, and αvβ8) recognize the tripeptide sequence arginine-glycine-aspartic acid (RGD) on latent activating peptide (LAP) of TGFβ [4–8]. Contractile cells, such as myofibroblasts, can bind to LAP-TGFβ through integrins and may activate latent TGFβ by exerting tensile force on the latent TGFβ complex [6, 9]. Blockade of αvβ6, for example, attenuates experimental fibrosis in several organs [10]. Integrin α8β1 also recognizes the RGD sequence on extracellular matrices and LAP-TGFβ [11, 12]. Developmental gene expression and histological analyses revealed high enriched ITGA8 expression in stromal cells of the lung such as pericytes and resident fibroblasts [13, 14]. Furthermore, histological examination of experimental fibrosis in murine lungs showed increased integrin α8β1 expression in fibrotic foci. Whether integrin α8β1 is functionally important in lung fibrosis is unknown.

ITGA8 knockout animals experience early postnatal mortality due to defects in nephrogenesis, which limits their utility in adult fibrosis models [2]. Targeted spatial deletions of ITGA8, however, do not result in developmental abnormalities seen in ITGA8 knockout mice. Studies of fibrosis in kidneys suggest ITGA8 signaling might attenuate renal fibrosis, but the functional role of ITGA8 in lung fibrosis has never been studied [15–17]. Since ITGA8 expression is restricted to stromal cells in the lung, we investigated the functional role of ITGA8 expression in myofibroblasts in lung fibrosis. Myofibroblasts are responsible for the main features encountered in the fibrotic focus. These cells deposit extracellular matrix and distort local architecture through cellular contraction. Platelet-derived growth factor receptor beta (PDGFRβ) is expressed in activated myofibroblasts across multiple organs, and in the lung, its expression overlaps extensively with alpha smooth muscle actin positive cells in fibrotic foci in mouse models of lung fibrosis [10, 18]. We leveraged advances in Cre-loxP transgenic mouse models to target spatial deletion of ITGA8 in PDGFRβ+ stromal cells and myofibroblasts.

## Materials and methods

### Mice

Animal protocols were approved by the University of Washington (UW) and Medical University of South Carolina (MUSC) Institutional Animal Care and Use Committees. Mice with the Pdgfrb-Cre transgene and floxed *Itga8* alleles were a gift from Dr. Dean Sheppard (UCSF). To generate study mice, *Itga8*^*flox/flox*^ females were crossed with Pdgfrb-Cre/+;*Itga8*^*flox/flox*^ or Pdgfrb-Cre/+;*Itga8*^*+/-*^ males. Both male and female mice at 8–12 weeks of age were used in this study. All mice were housed under specific pathogen-free conditions with food and water *ad libitum*. Mice were monitored daily following bleomycin administration for clinical signs of distress including dyspnea, hunched posturing, lethargy, rough hair coat, and poor grooming. Mice with >20% weight loss were monitored closely and euthanized if they met euthanasia criteria as approved by the Institutional Animal Care and Use Committees at the UW and MUSC.

### Bleomycin-induced lung injury

Transgenic mice underwent intratracheal instillation with 1.3 U/kg bleomycin (SICOR Pharmaceuticals, Inc, Irvine, CA) as previously described [19].

### Hydroxyproline assay

Left lungs from injured and uninjured mice were collected at day 21 following bleomycin administration. Left lungs were weighed and homogenized in 1mL deionized water. 100 μl of lung homogenate was mixed in 100 μl of 37% hydrochloric acid (Sigma #258148, St. Louis, MO) and heated at 120 degrees C for 3 hours. 50 μl of the acid hydrolyzed samples were added to 96-well flat bottom microplates in duplicates. Hydroxyproline content was then measured using the Hydroxyproline Assay Kit (Sigma #MAK0008, St. Louis, MO) per manufacturer’s protocol. Total protein content in the left lung homogenate was obtained by mixing 100 μl of lung homogenate with 100 μl of 2X protein lysis buffer. The sample was mixed and kept on ice for 30 min, with vortexing every 10 minutes. The samples were spun down and the supernatant was collected. Protein content in the protein lysate supernatant were detected by standard colorimetric assay using the Pierce^TM^ BCA Protein Assay Kit (Thermo Fisher Scientific #23225, Rockford, IL). Hydroxyproline measurements were then normalized to total protein content (expressed as μg hydroxyproline/mg total protein in left lung)

### Western blot

Equal amounts of protein were separated by sodium dodecyl sulfate-polyacrylamide gel electrophoresis (SDS-PAGE), and electrophoretically transferred to PVDF membrane. Membranes were blocked with 5% nonfat dry milk/0.05% Tween-20/PBS for 1hr at room temperature, incubated with primary antibodies (goat anti-ITGA8 antibody, 1:500, R&D Systems AF4076, Minneapolis, MN; mouse anti-GAPDH, 1:10,000, Millipore Sigma CB1001, Billerica, MA) overnight at 4°C, washed with 0.1% Tween-20/PBS, incubated with horseradish peroxidase-conjugated secondary antibodies (rabbit anti-goat IgG HRP, 1:10,000, Thermo Fisher Scientific #31402; goat anti-mouse IgG HRP, 1:10,000, Thermo Fisher Scientific #62–6520) for 1 hr, washed with 0.1% Tween-20/PBS and then developed with enhanced chemiluminescence (Pierce^TM^ ECL 2 Western Blotting Substrate, Thermo Scientific #80196).

### Isolation of lung PDGFRβ+ cells

Lungs were digested as described previously with minor modifications [13]. Mice were sacrificed with an overdose of isoflurane for dissection and digestion of lung tissue. Lungs were inflated with Liberase TL (0.2 mg/ml; Roche) and DNase I (0.1 mg/ml; Roche) in DMEM/F12. Inflated lungs were then explanted, minced, and incubated in digestion buffer with vigorous shaking for 45 min at 37°C. The suspension was filtered (40μm), centrifuged (300 x g, 10 minutes), and washed with DMEM/F12 containing 10% FBS. Cells were resuspended in complete Mouse Pericyte Medium (ScienCell, Carlsbad, CA, catalog #1231) and cultured in a T-75 flask coated with 0.2% gelatin until confluent. Cells were detached with Accutase (BD Biosciences, San Jose, CA), washed once with magnetic-activated cell sorting buffer (DPBS, 0.5% FBS, 2 mM EDTA, pH 7.2), and incubated with anti-CD45, anti-CD31, and anti-CD326 microbeads (Miltenyi, Auburn, CA) for 15 min for depletion of leukocytes, endothelial cells, and epithelial cells, respectively. Labeled cells were passed over a magnetized LD column (Miltenyi). Unlabeled cells were collected and incubated with PE-conjugated anti-PDGFRβ (Miltenyi, clone REA363). PDGFRβ+ cells were isolated by incubation with anti-PE microbeads and passage over a magnetized LS column (Miltenyi). Retained, PDGFRβ+ cells were collected and cultured in Mouse Pericyte Medium. Cells were used between passages 5 and 6.

### TGFβ stimulation

6 x 10^4^ cells per ml of purified PDGFRβ+ cells from Cre+ and Cre- animals were seeded into each well of 12-well tissue culture plates. Cells were serum starved overnight in base Mouse Pericyte Medium containing 0.1% BSA, insulin-transferrin-selenium (Gibco Life Technologies), 10 nM hydrocortisone (Sigma), and penicillin/streptomycin. Recombinant human TGFβ (R&D Systems, catalog #240-B) was added at a concentration of 10 ng/ml after overnight serum starvation. Vehicle (4 mM HCl, 1 mg/ml BSA) was used as the control. Cells were incubated for 24 hours before isolation of RNA.

### Cell adhesion assay

96-well plates were coated with 1 μg/ml recombinant mouse nephronectin (R&D Systems) or rat plasma fibronectin (Sigma-Aldrich) at 4°C. Control wells were coated with 1% BSA. Wells were washed with DPBS and blocked with 1% BSA in DPBS for at least one hour at 37°C. Purified PDGFRβ+ cells from Cre+ and Cre- animals were detached with Accutase and washed with DPBS, then were added to each well at a density of 4.7 x 10^5^ per ml in serum-free Mouse Pericyte Medium. Plates were centrifuged at 55 x g for 15 sec to evenly disperse cells. After 1 hour incubation at 37°C, plates were inverted to flick out unattached cells and media. Wells were washed once with DPBS and plates were centrifuged upside down at 55 x g for 15 sec. Attached cells were fixed and stained with 1% paraformaldehyde (PFA)/0.5% crystal violet for 30 min. Plates were washed in distilled H_2_O and dye was eluted from the cells with 10% acetic acid. Absorbance was measured at 595 nm and background adhesion on BSA was subtracted from each value.

### Confocal microscopy and histology

Right lungs were prepared as previously described[13]. Mouse lungs were perfused with 4% PFA and inflated to 25 cm H_2_O pressure for 15 min. Inflated lungs were then immersed in 4% PFA for 2 hours on ice, placed in 18% sucrose overnight at 4°C, embedded in OCT, snap frozen, and 10 μm cryosections were prepared. Primary antibodies against the following proteins were used for immunolabeling: PDGFRβ (1:100, Cell Signaling Technology #3169, clone 28E1, Beverly, MA), ITGA8 (1:100, R&D Systems AF4076). Lung sections were stained with hematoxylin and eosin (H&E) and picrosirius red. For semi-quantitative analysis of lung histology, each sample was evaluated by a blinded observer in 12 successive fields at 20X magnification. Fibrotic scoring as defined by Ashcroft et al. was applied to each field and averaged [20].

### Real-time PCR

Total RNA was isolated from cultured cells using PureLink RNA Mini Kit (ThermoFisher Scientific) in conjunction with DNase treatment as per manufacturers’ specifications. Total RNA was reverse-transcribed to cDNA using Applied Biosystems High-Capacity cDNA Archive Kit or iScript Reverse Transcription SuperMix (Bio-Rad, Hercules, CA). Real-time PCR was done using an ABI 7900HT or Bio-Rad CFX96 instrument with the use of pre-designed primers and probes (ABI TaqMan Gene Expression Assays). Quantification of gene expression was normalized to *Hprt* or *B2m* (endogenous controls). Pre-designed primers and probes include: *Acta2* (Mm01546133_m1), *B2m* (Mm00437762_m1), *Col1a1* (Mm00801666_g1), *Ctgf* (Mm01192933_g1), *Fn1* (Mm01256744_m1), *Hprt* (Mm03024075_m1), and *Itga8* (Mm01324958_m1). Analysis was performed in MS Excel calculating RQ by 2^-ΔΔCT^.

### BALF cytokines

Levels of CXCL1, CXCL2, CXCL10, MCP-1, and TNFα in undiluted cell-free BALF samples were assessed using a Milliplex Mouse Cytokine Magnetic kit (EMD Millipore Corp) according to the manufacturer’s instructions. Measurements were acquired and analyzed on a Magpix instrument with Milliplex Analyst 5.1 software (EMD Millipore Corp). Levels of total mouse TGFβ1 in undiluted, cell-free BALF samples collected at day 21 were assessed using DuoSet ELISA system (R&D Systems, DY1679-05) according to the manufacturer’s instructions.

### Lung mechanics

Mice were anesthetized with 5% isoflurane, and tracheotomies were performed by cannulating the tracheas with 18- or 19-gauge adapters, attached to a *FlexiVent* ventilator (SciReq, Montreal, Canada). Mice were ventilated at a tidal volume of 10 ml/kg body weight, a positive end-expiratory pressure of 3 cmH_2_O, and 150 breaths/minute (default). Spontaneous respiration was suppressed by intraperitoneal injection of pancuronium bromide (0.08 mg/kg) and maintenance of the mice on 2% isoflurane. Resistance (R) and Elastance (H) were determined using the Quick Prime-3 script and flexiWare software version 5.2.

Means of more than two groups of data were compared using one-way analysis of variance (ANOVA) for analysis of one independent variable or two way ANOVA, for analysis of two independent variables, followed by Tukey's honestly significant difference (HSD) post hoc test. We used Student T-test for comparison of paired parametric data and Mann-Whitney’s U test for non-parametric data. All tests were two-tailed and p values ≤ 0.05 were considered significant. Statistical analysis was performed using GraphPad Prism for Macintosh version 4.0c. All experiments were repeated with a minimum of 3 biological replicates and summary statistics are mean±SD or mean±SEM.

## Results

### Enhanced collagen upregulation in ITGA8-deleted PDGFRβ+ cells in response to TGFβ *in vitro*

**Fig 1:**
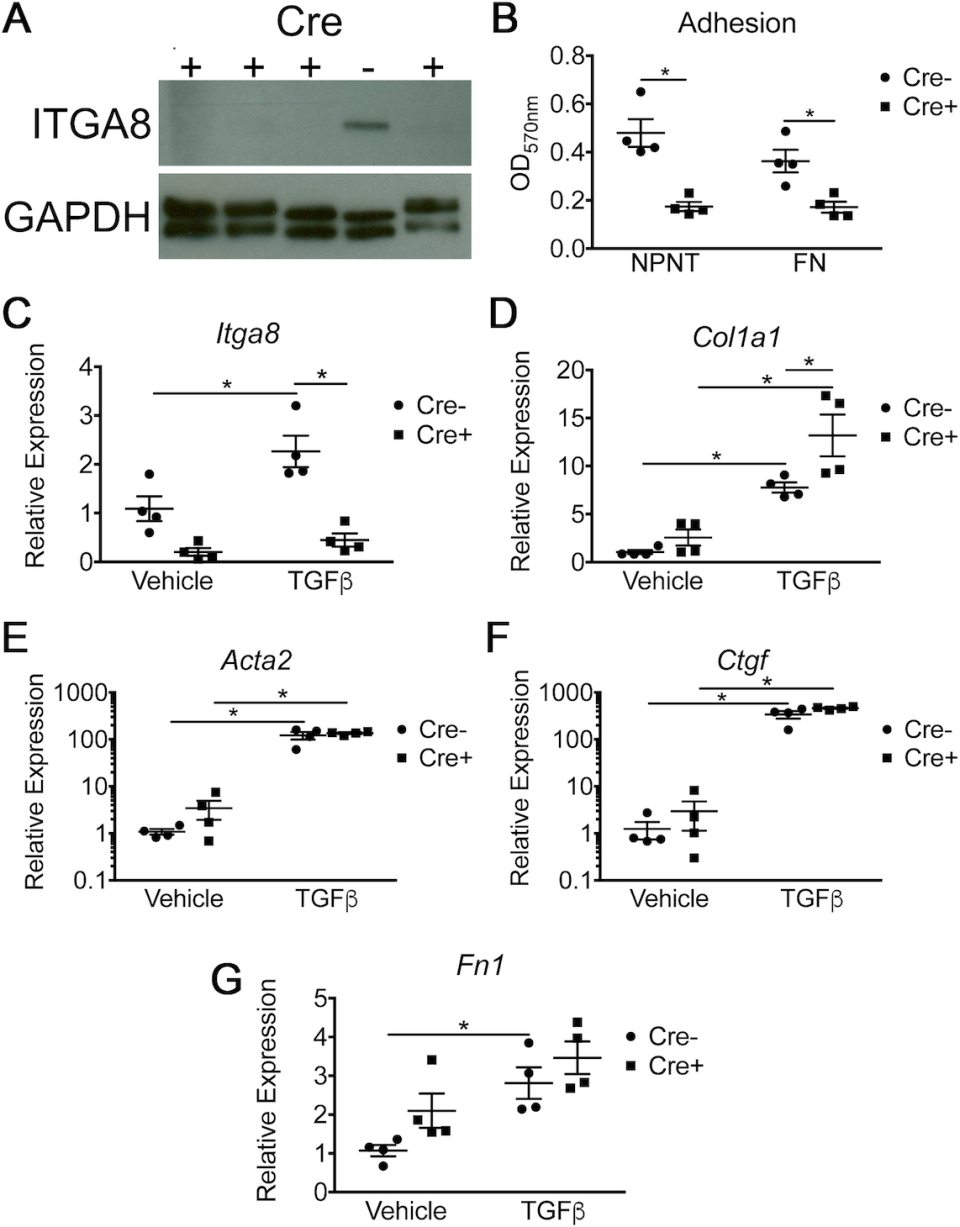
*In vitro* characterization of PDGFRβ-selected cells isolated from lungs of Pdgfrb-Cre+;*Itga8*^*flox/-*^ (Cre+) and WT or *Itga8*^*flox/flox*^ (Cre-) mice. (A) Western blot showing the absence of ITGA8 expression in Cre+ cells compared to Cre- cells. (B) Adhesion assay showing decreased binding to the substrates nephronectin (NPNT) and fibronectin (FN) by Cre+ cells. (C-G) PDGFRβ-selected cells were treated with TGFβ (10 ng/ml) or vehicle. mRNA expression is presented as fold change relative to Cre- in the untreated group (vehicle). Genes include: *Itga8* (C), *Col1a1* (D), *Acta2* (E), *Ctgf* (F), and *Fn1* (G). (*p<0.05, mean±SEM, n = 4).

Cultured PDGFRβ+ lung stromal cells from Cre+ transgenic mice showed decreased ITGA8 expression by Western blot and by qPCR compared to cells isolated from Cre- mice (Fig 1A and 1C). Cultured PDGFRβ+ cells from Cre+ animals demonstrated decreased adhesion to nephronectin (NPNT) and fibronectin (FN), two known α8β1 ligands, confirming functional knockdown of ITGA8 in Cre+ transgenic animals (Fig 1B). TGFβ treatment of PDGFRβ+ Cre- cells *in vitro* significantly increased *Itga8* gene expression but, as expected, did not change *Itga8* expression in PDGFRβ+Cre+ cells (Fig 1C). Cultured PDGFRβ+ cells from Cre+ animals treated with TGFβ showed increased *Col1a1* expression compared to cells from Cre- littermates by qPCR (Fig 1D). However, no difference was detected in other measures of fibrotic response including *Acta2*, *Ctgf*, and *Fn* by qPCR (Fig 1E–1G).

### Baseline effect of targeted ITGA8 deletion *in vivo*

**Fig 2:**
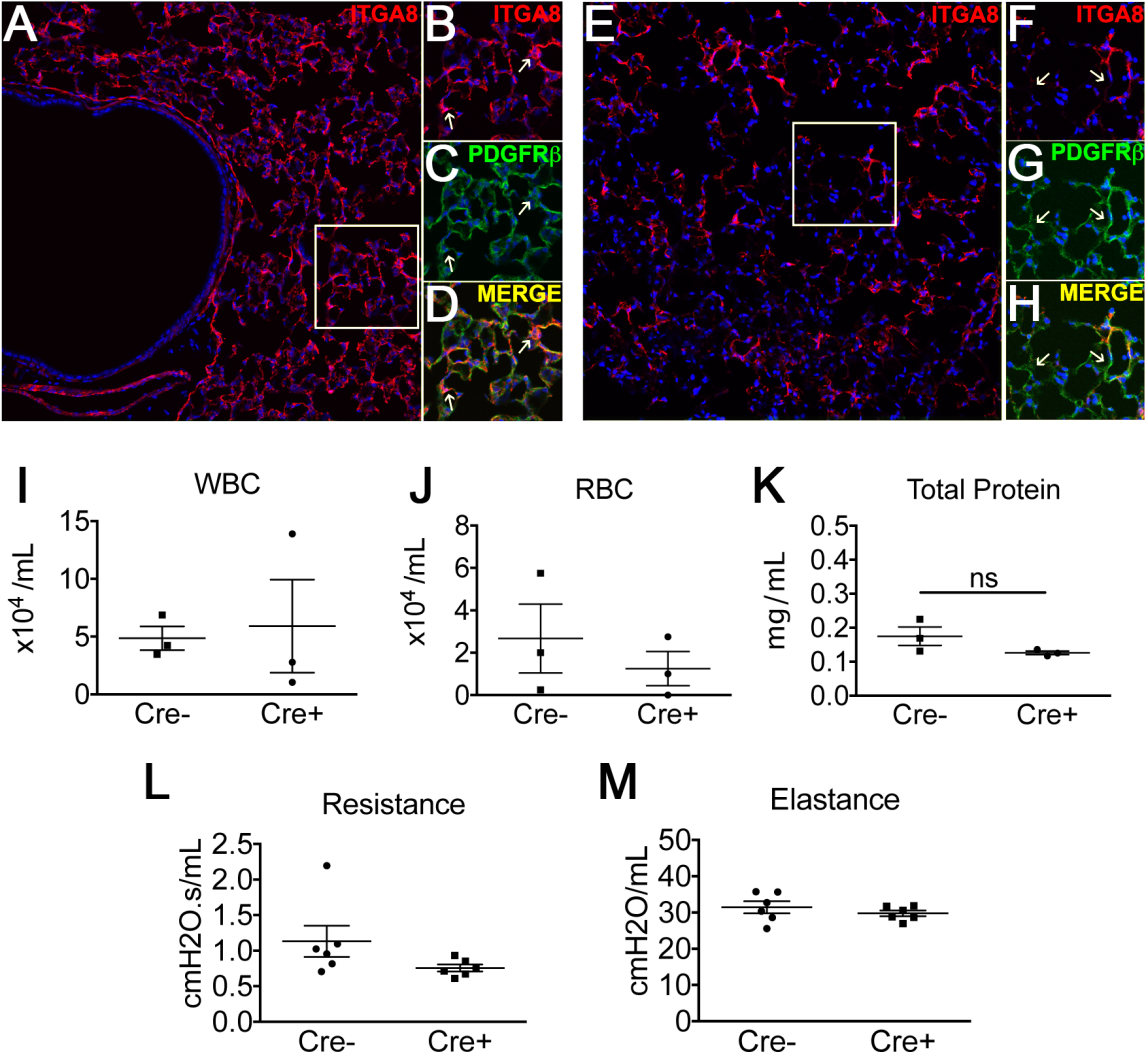
(A) Cre- animal stained for ITGA8 (red) at 20X. Magnified images of ITGA8 (B) and PDGFRβ (C) staining are shown in panels to the right. (D) Merged image shows co-staining of ITGA8 in PDGFRβ+ lung cells. (E) Cre+ animal showed markedly reduced ITGA8 staining (red). Magnified images of ITGA8 (F) and PDGFRβ+ (G) staining in Cre+ animals are shown in panels to the right. (H) Merged image shows reduced ITGA8 staining in PDGFRβ+ cells. (I-K) Analysis of BALF from Cre+ and Cre- mice revealed no differences in WBC, RBC, and total protein (mean±SEM, n = 3). (L,M) Baseline mechanics showed no difference in airway resistance and elastance (mean±SEM, n = 6).

Histological evaluation revealed high levels of ITGA8 expression in the adult murine lung at baseline whereas Cre+ animals demonstrated a significant knockdown of ITGA8 expression (Fig 2A–2H). Confocal images of lung sections co-staining for ITGA8 and PDGFRβ confirmed decreased ITGA8 staining in PDGFRβ+ cells from Cre+ animals (Fig 2A–2H). However, we observed ITGA8 expression in PDGFRβ- cells as well. When we examined Cre+ animals and littermates (Cre-) at baseline, we did not observe functional differences in their lungs in the absence of injury. Baseline BALF analysis revealed no significant differences in cell counts, total BALF protein, or hydroxyproline content (Fig 2I–2K). Physiologic measurements including lung elastance and airway resistance were also comparable between Cre+ animals and littermates at baseline (Fig 2L and 2M). Therefore, in the absence of injury, targeted deletion of ITGA8 in PDGFRβ+ cells of murine lungs did not lead to observable phenotypic differences compared to wildtype littermates.

### Effect of targeted ITGA8 deletion on early inflammatory response in the bleomycin model

**Fig 3:**
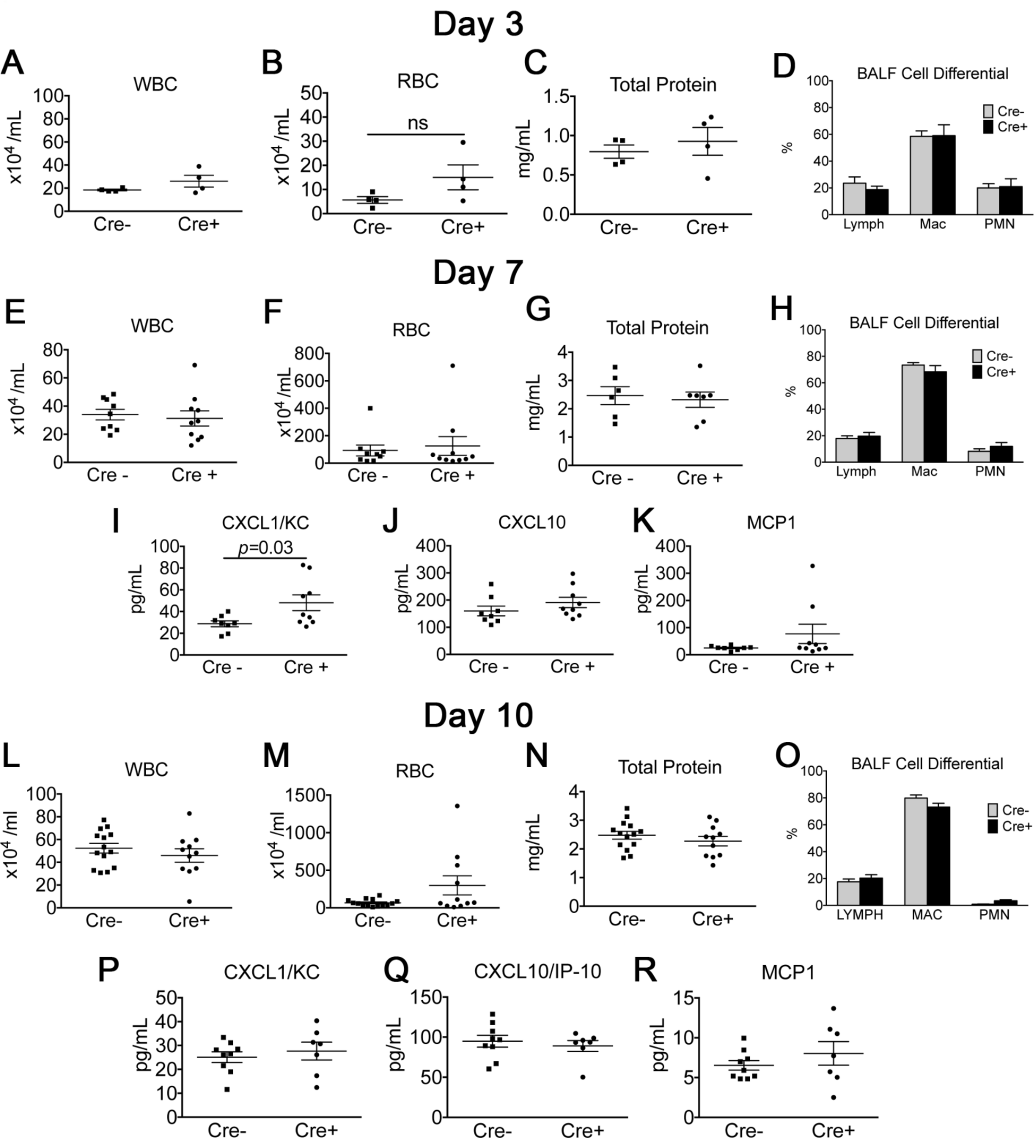
Evaluation of lung inflammatory response at days 3, 7, and 10. (A-D) BALF collected from Cre+ and Cre- mice at day 3 following bleomycin administration revealed no significant difference in BALF WBC, RBC, total protein, or cell differential. (E-K) BALF collected from Cre+ and Cre- mice at day 7 following bleomycin administration revealed no significant difference in BALF WBC, RBC, total protein, and cell differential. Measurements of select inflammatory cytokines in BALF revealed elevation of CXCL1 in Cre+ compared to Cre- animals but no difference in CXCL10 or MCP-1 (mean±SEM, n≥7). (L-R) BALF analysis at day 10 following bleomycin induced lung injury revealed no significant difference in BALF WBC, RBC, total protein, cell differential, or levels of CXCL1, CXCL10, and MCP-1 (mean±SEM, n≥7).

Because the effect of ITGA8 deletion has never been tested in a lung injury model, we first evaluated whether differences in acute inflammation existed with bleomycin challenge, which may confound the interpretation of fibrosis in the repair phase. Cre+ and Cre- mice were given bleomycin by intratracheal instillation and were harvested at days 3, 7, and 10. Examination of the BALF revealed no significant differences in white and red cell count, total protein, nucleated cell count, and cell differential (Fig 3A–3H and 3L–3O). We further examined a panel of pro-inflammatory cytokines in the BALF. At day 7, there was a significant elevation in CXCL1 in the Cre+ group (Fig 3I). No difference was observed between Cre- and Cre+ groups for BALF CXCL10 or MCP-1 (Fig 3J and 3K and 3P–3R). CXCL2 and TNFα levels were undetectable for both Cre- and Cre+ groups in the BALF on days 7 and 10 (data not shown). Although we observed elevated CXCL1 levels in the Cre+ group at day 7, we did not observe significant differences compared to the Cre- group in other inflammatory cytokines tested. Moreover, other parameters of inflammation including BALF cell counts, the percentage of PMNs in BALF, and total protein were similar between Cre- and Cre+ groups at day 7, suggesting the difference observed in BALF CXCL1 did not lead to a significant phenotypic difference in bleomycin-induced acute inflammation between Cre- and Cre+ animals.

### Effect of targeted ITGA8 deletion in bleomycin-induced fibrosis

**Fig 4:**
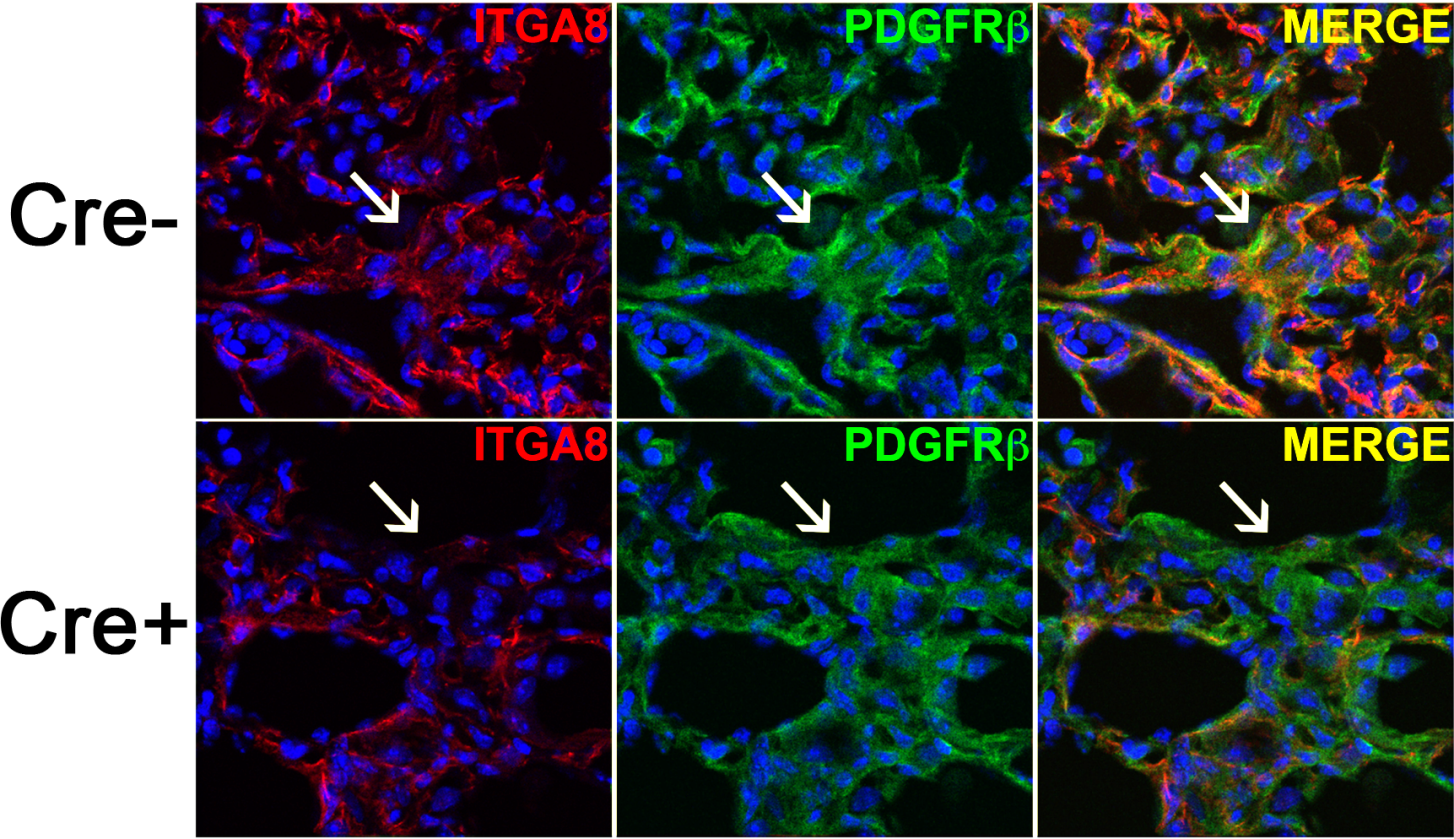
Confocal image of fibrotic region in Cre- and Cre+ mice co-staining for ITGA8 and PDGFRβ illustrating diminished ITGA8 staining in PDGFRβ+ fibrotic focus in Cre+ mice (arrow).

**Fig 5:**
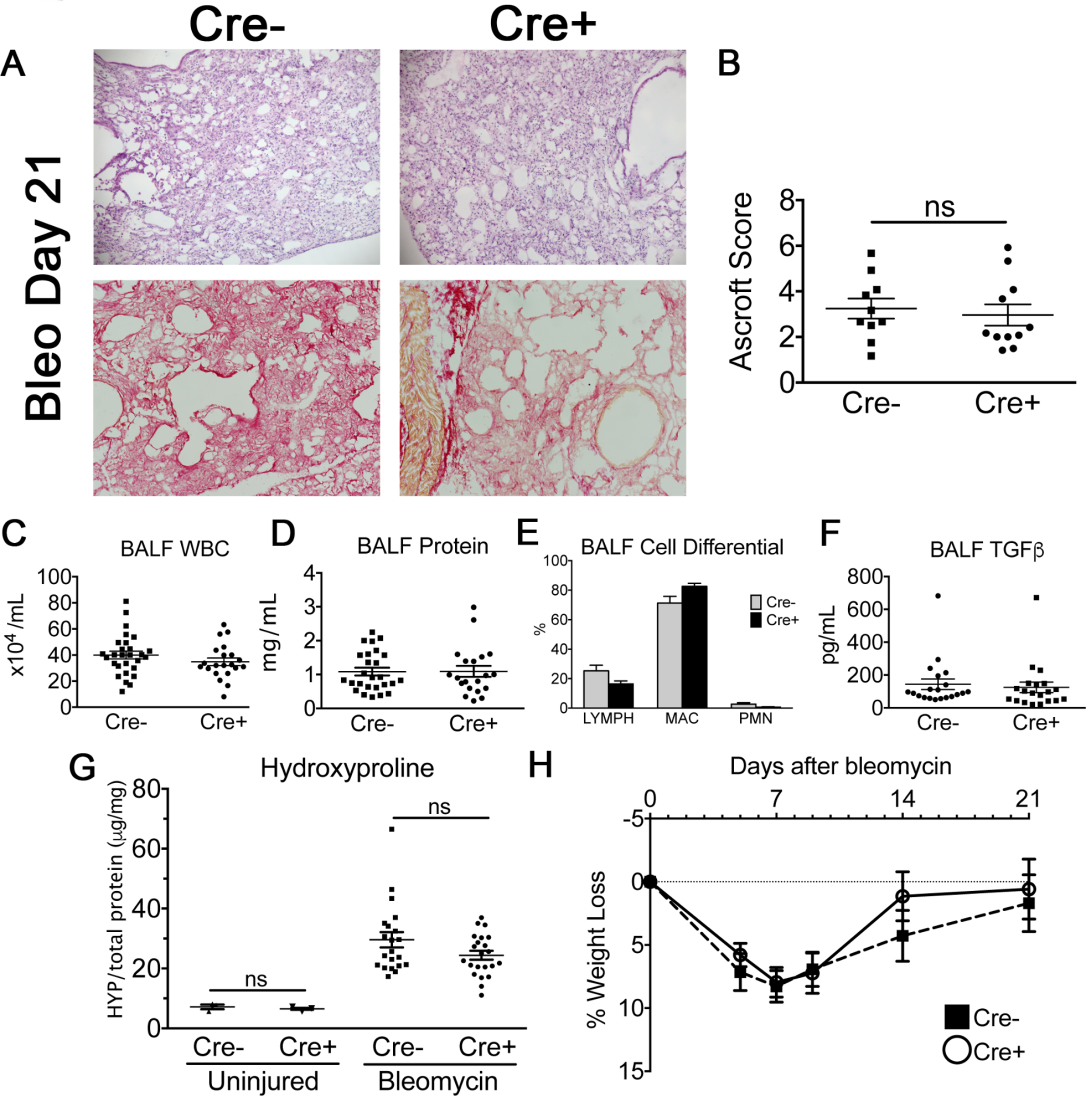
(A) Representative H&E (top) and picrosirius red (bottom) images of lung sections (20X) from Cre- and Cre+ mice at 21 days after bleomycin administration. (B) Ashcroft histopathology scoring of mouse lung sections at 21 days (mean±SEM, n≥10). (C-E) BALF WBC, total protein (mean±SEM, n≥28), and cell differential (mean±SEM, n≥11) were similar between Cre- and Cre+ animals at day 21. (F) Total TGFβ measurement in BALF at day 21 by ELISA (mean±SEM, n = 20). (G) Hydroxyproline measurements in Cre- and Cre+ animals in uninjured mice and in bleomycin injured mice at day 21. Hydroxyproline measurments in uninjured mice revealed no differences in baseline lung collagen content (mean±SEM, n = 3). Both Cre- and Cre+ showed significantly higher hydroxyproline content compared to uninjured Cre- and Cre+ animals (*p<*0.05), but no significant difference was observed between Cre- and Cre+ bleomycin injured mice at day 21 (mean±SEM, n≥28). (H) Average percentage weight loss of Cre- and Cre+ mice following bleomycin lung injury (mean±SEM).

Based on our *in vitro* results, we speculated Cre+ animals would have an exaggerated fibrotic response in the bleomycin lung injury model, given ITGA8 knockdown led to increased collagen expression in TGFβ-stimulated PDGFRβ+ cells. Cre+ and Cre- mice were exposed to bleomycin (1.3U/kg mouse) by intratracheal instillation and harvested at 21 days. Under fluorescent microscopy, peribronchiolar regions of fibrosis in Cre- animals showed abundant PDGFRβ and ITGA8 co-staining on confocal imaging (Fig 4). In contrast, Cre+ animals showed significantly diminished ITGA8 staining in PDGFRβ+ regions of fibrosis. Despite measurable ablation of ITGA8, there were no obvious differences between the genotypes in lung histology, leukocyte counts, total protein, cell differential, total TGFβ1 in the BALF (Fig 5A–5F). Biochemical analysis of collagen accumulation at day 21 by left lung hydroxyproline content did not reveal significant difference between Cre- and Cre+ animals (Fig 5G). Weight loss following bleomycin administration was similar between the two groups (Fig 5H).

## Discussion

Previous studies of organ injury and fibrosis in rat and mouse models have shown expression of ITGA8 in vascular smooth muscle cells and stromal cells in lung, liver, and kidney during homeostasis and following injury [3, 21]. However, exploration of the functional relevance of ITGA8 expression in organ fibrosis has been limited by embryonic or early postnatal mortality encountered in ITGA8 global knockout animals, presumably due to abnormal renal development [2, 22]. In this study, we used mice harboring floxed alleles of Itga8 and expressing Cre recombinase from the PDGFRβ promoter to ablate integrin α8 in PDGFRβ+ stromal cells and myofibroblasts. We showed that this animal model achieved near total knockdown of ITGA8 in lung PDGFRβ+ cells at the mRNA and protein level, leading to reduced adhesion of these cells to known integrin α8 substrates *in vitro*.

*In vitro* stimulation of PDGFRβ+ cells with the pro-fibrotic cytokine TGFβ produced increased expression of collagen I, α-smooth muscle actin, CTGF, and fibronectin, demonstrating that these cells are key responders in fibrosis. However, in Cre+ cells, mRNA levels of collagen were elevated over that in Cre-, consistent with findings of increased renal fibrosis in ITGA8 knockout animals [17]. Furthermore, there was suggestion of increased *Acta2* expression in the absence of TGFβ stimulation, even though the difference did not reach statistical significance. A similar finding was reported for cells in the glomerulus of ITGA8 global KO kidneys [23, 24]. Thus, ITGA8 signaling may attenuate activation of PDGFRβ+ cells *in vitro*, as seen previously in renal fibroblasts [15]. Based on these *in vitro* observations, we speculated that lack of ITGA8 expression in lung PDGFRβ+ stromal cells would exacerbate the pro-fibrotic response following injury. We therefore tested this hypothesis in mice with Pdgfrb-Cre and floxed alleles of Itga8 in bleomycin-induced lung fibrosis.

We first characterized the transgenic animals with ITGA8 deletion in PDGFRβ+ cells at baseline, without any injury. We found no evidence of abnormal lung physiology in homeostasis, as measured by lung function, histology, and BALF chemistries and cell counts. The bleomycin model does not lead to differences in the acute inflammatory response between Cre+ and Cre- animals as BALF leukocyte cell count, levels of proinflammatory cytokines, and total protein at days 3, 7, and 10 were not significantly different between Cre+ and Cre- animals. While the *in vitro* data suggested ITGA8 deletion may lead to increased collagen I production during lung fibrosis, we found no differences in hydroxyproline levels between Cre+ and Cre- transgenic animals in the bleomycin model. Based on these data, we conclude that ITGA8 deletion from PDGFRβ+ stromal cells does not affect lung fibrosis in the bleomycin model. Furthermore, ITGA8 deletion does not alter homeostatic functions of the lung nor the acute inflammatory response in this model.

Itga8 global KO mice have been used to study the role of this integrin subunit in fibrosis of the kidney and heart, with the caveat that surviving mice display renal mass and physiology that are different from WT controls. In the kidney, lack of ITGA8 worsens tubulointersititial fibrosis [17, 23, 25] and delays healing in a model of glomerulonephritis [23]. The latter study controlled for the decreased renal mass in the KOs by performing a uninephronectomy in the WTs. In the heart, however, global deletion of ITGA8 does not change the extent of hypertension-induced fibrosis in KO mice, despite the upregulation of ITGA8 in in this disease model [25]. Although to our knowledge no one has assessed lung fibrosis in the global KO, our results show that ITGA8 in PDGFRβ+ cells is dispensable in the bleomycin model.

There are several important limitations to our study. First, this transgenic model only tested spatial deletion of ITGA8 in PDGFRβ+ cells. Functional relevance of ITGA8 expression in other cell types during fibrosis was not assessed. Other conditional knockouts will need to be generated to address the question. Second, while the *in vitro* data are consistent with previous findings that ITGA8 knockout led to worse tubulointerstitial fibrosis, there was no significant difference in bleomycin-induced lung fibrosis. As suggested by Hartner et al. for kidney fibrosis [17], ITGA8 may play a more central role in less severe forms of chronic fibrosis than that seen in the bleomycin model. While knockdown of integrin α8 is substantial in our transgenic, a low level of expression may be sufficient to mask the effects of ITGA8 deletion. Finally, although we observed no significant changes in the expression of other alpha integrins in isolated PDGFRβ+ cells (unpublished data), we cannot rule out the possibility that one or more of the alpha chains compensates for the lack of ITGA8 in our model in vivo.

In summary, while ITGA8 deletion resulted in increased collagen I production in cultured PDGFRβ+ cells, ITGA8 deletion in PDGFRβ-Cre;ITGA8^flox/flox^ transgenic animals did not result in significantly worse fibrosis compared to Cre- littermates in the bleomycin lung injury model. Though ITGA8 exhibits LAP-TGFβ binding similar to other alpha integrins in the family, expression of ITGA8 in PDGFRβ+ cells does not appear to have a major biological role in lung fibrosis.

## Supporting information

S1 Dataset PLOS One dataset—Raw data for the figures in this report. (XLSX)
